# Pennsylvania

**Published:** 1895-05

**Authors:** 


### Pennsylvania.

Efforts are being made in the present session of the State Legislature of Pennsylvania to extend the provisions of the Milk Act of 1885, now applying to cities of the second and third class, to cities of the first class. This act makes the purveyor of milk liable for the acts of his employés in any violation of the provisions of the act. It demands a standard of 12.5 per cent. milk solids, not less than 3 per cent. of which shall be fat, with a specific gravity of 1029 to 1033, at a temperature of 60° Fahrenheit.

The bill establishing a State Board of Veterinary Medical Examiners in Pennsylvania passed second reading on special order on April 8th, and on the 9th passed third reading by the overwhelming vote of 147 to 11, in the House, where it was ably managed and well championed by Representative Woodring, of Northampton County, to whom the veterinary profession of Pennsylvania owe a debt of gratitude. It is now in the Senate.

We inadvertently published in the April number the statement that the Live Stock Sanitary Board bill had passed the Senate. It has only reached first reading in the Senate, having passed the House by a large majority. All veterinarians and their friends want to give this bill their close attention, as the session of the Legislature is fast drawing to a close.

